# Estimating Clinical Manifestations in Cell Biology Teaching is a Suitable Active Element for Teaching Beginning Medical Students

**DOI:** 10.1177/23821205261478406

**Published:** 2026-09-02

**Authors:** Pavel Dvorak, Zbynek Tonar

**Affiliations:** 1 Department of Biology, Faculty of Medicine in Pilsen, Pilsen, 37740 Charles University, Czech Republic; 2 Biomedical Center, Faculty of Medicine in Pilsen, Pilsen, 37740 Charles University, Czech Republic; 3 Department of Histology and Embryology, Faculty of Medicine in Pilsen, Pilsen, 37740 Charles University, Czech Republic

**Keywords:** active learning, diagnostic reasoning, cell biology, genocopy, clinical manifestations, leukocyte extravasation, muscular dystrophies

## Abstract

**Objective:**

Cell biology courses in medical education often emphasize molecular detail without sufficient clinical integration, which can reduce student motivation and hinder application of knowledge. Embedding diagnostic reasoning into basic science teaching may enhance engagement and relevance. We developed short, task-based exercises in which students are actively encouraged to infer potential clinical symptoms of genetically determined diseases from cellular mechanisms.

**Methods:**

Each task followed three steps: (1) introduction to a specific cellular process, (2) guided prediction of clinical manifestations resulting from dysfunction of key proteins, and (3) verification of hypotheses through published literature. Tasks were implemented into first-year medical student seminars of Medical Biology in both face-to-face and online formats. Evaluation was based on assessments by experienced teachers and a student questionnaire at the end of the course.

**Results:**

Two illustrative tasks were presented: role of adhesion proteins in leukocyte extravasation, linked to leukocyte adhesion deficiency syndromes, and sarcolemma–cytoskeleton interactions, associated with various muscular dystrophies. Teachers as well as students consistently rated the tasks positively in surveys conducted. Better understanding of the connection between cellular mechanisms and clinical symptoms, greater confidence in diagnostic reasoning, and an increased ability to search and interpret scientific literature were reported. Based on the evaluation, modifications to the learning outcomes were proposed to place greater emphasis on the ability to draw conclusions from cellular processes.

**Conclusion:**

Integrating short, clinically anchored exercises into cell biology teaching provides an effective means of activating student learning and fostering early diagnostic reasoning. This approach strengthens the perceived relevance of basic science, supports critical thinking, and offers a scalable model for enhancing preclinical medical curricula.

## Introduction

Modern medical school curricula increasingly recognize that students learn most effectively when foundational sciences are taught in ways that are connected, human, and intellectually engaging. Rather than simply transmitting facts, contemporary teaching emphasizes understanding processes that reveal how cellular mechanisms translate into clinical manifestations. Recent reviews underscore wide institutional variation in approaches to teaching clinical reasoning during the preclinical years and note that students value clarity, accessibility, and meaningful integration of content.^1^

Many programs therefore pursue deeper interconnection between foundational and clinical subject matter. Studies of co-teaching by clinicians and basic scientists suggest that this approach can raise students’ motivation and sharpen their appreciation of the clinical relevance of cellular mechanisms.^2^ Team-based integrated sessions likewise tend to feel more coherent and applicable from the learner’s point of view.^3^ Large seminar formats that embed real clinical cases also show that combining disciplinary perspectives helps novices build knowledge and confidence in emerging diagnostic reasoning.^4^

Active learning strategies are not merely supplements to lectures; used well, they are powerful tools for deeper understanding. By engaging students in formulating hypotheses, comparing arguments, testing conclusions, and learning from mistakes, these methods cultivate the habits that underlie clinical reasoning.^5^ Systematic reviews further indicate that structured tasks focused on diagnostic thinking can start shaping reasoning early in training.^6^ Embedding literature search and interpretation reinforces critical appraisal and the habits of evidence-based practice.^7^

In this context, we designed concise, clearly structured tasks that link cell biology with clinical reasoning. Students first gain an understanding of a cellular mechanism, then predict possible clinical manifestations arising from its disruption, and finally verify their hypotheses using published evidence. The sequence fosters analytical thinking and information literacy while illustrating that distinct genetic or molecular defects may converge on overlapping clinical presentations—a phenomenon frequently encountered in clinical practice.

## Materials and Methods

### Educational Design

The teaching intervention was designed according to the principles of constructive alignment and backward design.^8^ The intended learning outcomes were that students should (1) understand key cellular mechanisms and their interconnections with processes at the level of the whole human body, (2) infer potential clinical symptoms arising from protein dysfunction within those mechanisms, and (3) verify their inferences by consulting professional literature. Learning activities and assessments were directly aligned with these outcomes through the design of short, structured tasks.

### Task Structure

Each task followed a **three-step sequence**:

**1. Concept introduction:** Students were introduced to the basic components and functioning of a selected cellular process (e.g., leukocyte extravasation, sarcolemma–cytoskeleton interactions). Teaching materials included concise reading assignments or short preparatory lectures.
**2. Symptom inference:** Students were actively encouraged to predict possible clinical manifestations of congenital diseases that could arise from loss of function of one or more proteins within the process. This stage emphasized group discussion, hypothesis generation, and diagnostic reasoning.
**3. Verification through literature:** Students searched for relevant information in scientific publications or databases to confirm or refine their predictions. Teachers guided students in distinguishing reliable sources and interpreting clinical data.

This design ensured that students not only gained knowledge of cellular biology but also practiced its application in diagnostic reasoning.

### Implementation

The new tasks were incorporated into first-year medical seminars at the Faculty of Medicine in Pilsen, Charles University, Prague, Czech Republic. Specifically, they were implemented in the teaching of 2 groups (15 students each) of the General Medicine field (out of a total of 300 students) and 1 group (15 students) of the Dental Medicine field (out of 60 students) led by the authors of this work. The groups were not selected according to any special criteria and therefore did not differ in composition from other groups in the same academic year. This innovation was first included in the teaching in the 2022/2023 academic year, and then in all subsequent years until the present, i.e. three academic years to the present.

Each seminar group formed a dedicated team on the MS Teams platform, which facilitated distribution of preparatory materials, sharing of resources, and discussion beyond class hours.

• **Step 1 (concept introduction)** was conducted as self-study with provided resources.
• **Step 2 (symptom inference)** was carried out during teacher-led seminars, typically using small-group discussions.
• **Step 3 (literature verification)** was completed either within the seminar or as homework, with findings shared and evaluated in the next session.

The approach was used in both **face-to-face** and **distance-learning** formats, demonstrating flexibility and scalability.

### Examples of Tasks

Two illustrative tasks were developed and implemented:

• **Leukocyte extravasation and immunodeficiency:** Students predicted the consequences of impaired adhesion proteins, leading to discussion of leukocyte adhesion deficiency syndromes.
• **Sarcolemma–cytoskeleton connections and muscle stability:** Students considered how loss of structural proteins could cause membrane fragility, linking their reasoning to muscular dystrophies.

Each example highlighted how distinct molecular dysfunctions in the same process can result in **convergent clinical manifestations**, reinforcing the concept of *genocopy*.

### Nature of Study and Evaluation

This was a descriptive report on educational innovations with retrospective evaluation by experienced teachers and routinely collected anonymous feedback from students. The presented work was not designed as a randomized controlled trial or a comparative effectiveness study.

The evaluation of the teaching innovation we describe was based primarily on observation and long-term experiences of the participating teachers. Student perceptions of the tasks were evaluated using anonymous **end-of-course surveys** routinely administered in all seminars (Supplementary Material 1). Surveys included both structured items and open comments on strengths and weaknesses of the course. For this study, responses were analyzed semi-quantitatively.

This work adheres to Standards for QUality Improvement Reporting Excellence (SQUIRE 2.0) guidelines for quality improvement in health care^9^; the completed checklist is included in the supplemental materials (Supplementary Material 2).

## Results

### Example No. 1 – The Importance of Cell Adhesion During Leukocyte Extravasation

#### Learning Outcomes

After solving this task, students know the importance of selectins and integrins (belonging to adhesion proteins on the surface of human cells) during leukocyte migration. Students understand why loss of function of these adhesion proteins disrupts leukocyte capture, adhesion, or transmigration. Using the example of leukocyte extravasation, they can explain the relationship between the dysfunction of several different proteins and the identical clinical manifestations of diseases known as Leukocyte adhesion deficiency (LAD) syndromes - reduced immune response, recurrent infections, delayed wound healing, and abnormal leukocyte counts. Students also gained the knowledge, how to search for and correctly interpret information from different published sources.

#### Background Information

The process by which various types of leukocytes (primarily neutrophils, monocytes, and lymphocytes) pass through the vessel wall to the site of inflammation (leukocyte extravasation) is one of the basic mechanisms of the innate immune response. This process is induced by the secretion of signaling proteins (chemokines) produced, for example, by the penetration of a pathogenic microorganism or mechanical tissue damage.^10^ For educational purposes, this continuous immune process is conveniently divided into individual subphases, which represent a series of different adhesive and signaling interactions. The subphases are usually defined – capture (tethering), rolling, arrest (adhesion), crawling (locomotion), and transendothelial migration (diapedesis) that can occur by crossing either endothelial cell contacts, or more rarely through pores in the body of endothelial cells.^11^ Vascular endothelial cells are stimulated to express various types of adhesion proteins, whose interactions with ligands and receptors on the leukocyte surface are crucial for the successful course of the entire process. At the beginning of this process, the most important role is played by interactions between selectins and their ligands, an example of which is the interaction of P-selectin on the endothelial cell surface and P-selectin glycoprotein ligand 1 (PSGL1) on the leukocyte surface. Gradually, the so-called leukocyte activation occurs, i.e. modification of other ligands and receptors on the leukocyte surface so that they are prepared for functional interactions. Integrins, immunoglobulins and their receptors, and a number of other adhesion and signaling proteins occurring in intercellular junctions play a crucial role for the successful completion of the process. Examples of cooperation between integrins include the interactions between intercellular adhesion molecule 1 (ICAM1) and lymphocyte function-associated antigen 1 (LFA1) or vascular cell-adhesion molecule 1 (VCAM1) and very late antigen 4 (VLA4).^12^ Some relationships between specific molecules are more general, and other relationships are specific to specific types of leukocytes. The process is also influenced by other proteins that mediate cooperation with changes in the cell cytoskeleton.

#### Questions on Inferring Clinical Manifestations

1) Which proteins play a key role in the penetration of leukocytes through the vessel wall towards the site of inflammation, so-called leukocyte extravasation?
2) Which subphases of this process would be disrupted by the loss of function of the given proteins?
3) If an individual were born with a congenital loss or significant limitation of the function of any of the given proteins, what overall clinical manifestations would you expect?

#### Expected Answers

If the function of selectins or their ligands, typical for leukocyte interactions with endothelial cells, is lost or significantly reduced, the beginning of the entire process, i.e. the capture/tethering and rolling subphase, will be disrupted. If the interaction between specific integrins, immunoglobulins and their receptors is disrupted, leukocyte activation, their stronger adhesion to endothelial cells or the final penetration (diapedesis) will be affected. We expect students to realize that a strong impairment of this process can generally result in an innately reduced or delayed immune response to infection or tissue injury. Patients with such diseases could experience more severe infections and injuries and slower healing from birth. Students could possibly also mention that such patients could have an increased level of leukocytes in the blood and, conversely, a reduced formation of pus at the site of inflammation.

#### Questions to Verify Putative Clinical Symptoms and Diseases

1) Can you verify whether diseases with impaired immunity due to congenital disorders of the leukocyte extravasation process have actually been recorded in humans?
2) Which characteristic clinical manifestations are mainly mentioned in the literature?
3) Compare the information you found with your assumptions. What were the differences and similarities?

#### Expected Answers

We assume that students will begin by searching for information on a freely available Internet search engine. The lecturer may also appropriately direct them to more specialized publicly available database resources, or the entire task may be preceded by a closer acquaintance with how to distinguish quality specialized Internet resources and where to look for them. Even students using a regular Internet search engine should find information about the existence of a group of diseases known as leukocyte adhesion deficiency (LAD) syndromes. The depth of more detailed information about this group of diseases depends on the specific requirements of the given course.

These are rare autosomal recessive diseases, however, research into them has been crucial for understanding the function of many cellular proteins. A common clinical symptom is repeated, difficult-to-treat bacterial and fungal infections, most prominent in the skin and mucosal surfaces. A frequent common manifestation is also persistent neutrophilia without an obvious infectious cause and a dramatic increase in myeloid leukocytes in the blood during infections (up to 20 times above normal white blood cell count values). Individual syndromes also show other specific symptoms and variability in phenotype can be found even within these individual syndromes.^13^ The cases described in the literature so far can be explained on the basis of variations in genes, which result in a disruption in the interactions between leukocyte-specific integrins or selectins and their ligands.^14^ The only treatment with curative potential is hematopoietic stem cell transplantation.

#### Additional Questions and Information

The discussion within this task can be appropriately expanded with other questions, such as the question: Which injury is among the first injuries in each of our lives? It is the healing of the umbilical stump after cutting the umbilical cord after birth. The umbilical cord typically separates from a newborn within 5 to 15 days after birth, though the normal range can extend up to 3 weeks. As it dries, the stump will change in color from yellowish-green to brown to black before naturally falling off.^15^ Significantly slower healing may be the first symptom of diseases with congenitally reduced immunity.

### Example No. 2 – The Importance of the Connection Between Cytoskeleton and Extracellular Matrix in Muscle Cells

#### Learning Outcomes

By solving this problem, students have an idea of the mechanism ensuring the stability of the cytoplasmic membrane of muscle cells, which is subjected to enormous mechanical pressure. They can name some key proteins for this purpose such as dystrophin and laminin. Students reason that impaired links between the sarcolemma, cytoskeleton, and extracellular matrix cause membrane fragility, necrosis, and progressive weakness. They know that these inferences match the pathology of Duchenne and Becker muscular dystrophies, as well as laminin- and collagen-related congenital dystrophies. Literature research further reinforced understanding of how diverse protein defects can manifest with comparable clinical symptoms.

#### Background Information

Muscle cells (myocytes) are specialized cells capable of active contraction and ensuring, among other functions, the movements of internal organs and the entire human body. Skeletal muscle fibers and cardiac muscle cells in particular must constantly cope with the mechanical pressure on their cytoplasmic membrane (sarcolemma), which is created during their activity. The problem of excessive pressure on the sarcolemma was solved by distributing its effect on other cellular and extracellular structures. The sarcolemma is connected to the cytoskeleton fibers in the direction of the cell, and in the opposite direction it is connected to the network structure of the extracellular matrix.^16^ Actin microfilaments in particular, but also tubulin microtubules and keratin intermediate filaments are connected to the complex of glycoproteins located in the sarcolemma via the specialized protein dystrophin.^17^ Its counterpart on the outside can be considered the protein laminin, which allows for connection to collagen fibers, of which collagen types VI and IV are indispensable for maintaining the integrity of muscle fibers and their regeneration.^18^

#### Questions to Infer Clinical Manifestations and Diseases

1) How is it ensured in human muscle cells that their cytoplasmic membrane is not subjected to too much pressure during muscle contraction?
2) Which proteins would you consider to be key for this task?
3) What consequences at the cellular level could be the interruption of the connection of the sarcolemma of muscle cells with cytoskeletal fibers or extracellular matrix components?
4) What clinical manifestations would you expect in patients with an inborn defect in the function of proteins that are key to this connection?

#### Expected Answers

The strong pressure exerted on the cytoplasmic membrane (sarcolemma) by muscle cell contraction is transmitted to other fibrous structures surrounding the cytoplasmic membrane via junctional elements. Key proteins for these junctions include dystrophin, laminin, fibronectin, components of the glycoprotein complex spanning the sarcolemma (dystroglycan, syndecan, and integrin), and some types of collagen. We expect students to conclude that excessive mechanical pressure on the sarcolemma of muscle cells, which is not further distributed to other cellular components, can cause mechanical damage (cracks to ruptures) of the sarcolemma. Mechanical disruption of the cytoplasmic membrane of cells is generally such a fundamental damage to the internal environment of the cell that it usually causes cell death in the form of necrosis. Given the specialization of muscle tissue, its regeneration is a more complex process based on the activation of muscle stem cells. Under permanently deteriorated conditions of the stability of the internal cellular environment, functional muscle tissue is usually replaced by fibrotic tissue.^19^ With an increasing proportion of fibrosis, the function of the given muscle decreases, the muscle weakens and eventually stops functioning completely. A common feature of such diseases could therefore be described as progressive muscle damage and weakness.

#### Questions to Verify Alleged Clinical Symptoms

1) Can you find convincing information about the existence of actually described cases of human diseases caused by congenital dysfunction of proteins involved in connecting the sarcolemma with the cytoskeleton or components of the extracellular matrix?
2) Which characteristic clinical manifestations are mainly mentioned in the literature?
3) Compare the information you found with your assumptions. What were the differences and similarities?

#### Expected Answers

We assume that students will primarily find information about Duchenne and Becker muscular dystrophy, caused by a non-functional or only partially functional protein dystrophin. For the intended purpose of our task, it is important that students also find out information about the existence of other described muscular dystrophies, whose pathophysiology has an identical mechanism. These diseases include congenital muscular dystrophies arising from pathogenic variants within the laminin alpha-2 chain gene (LAMA2; merosin), type VI collagen genes (COL6A1, COL6A2, and COL6A3), or a number of genes whose proteins are essential for the functioning of the cooperating glycoprotein complex in the sarcolemma.^20^ The depth of information about the diseases discussed again depends on the specific requirements of the course.

#### Additional Questions and Information

The topic presented in the second example allows, among other subtopics, for a discussion of the largest human gene DMD and most modern methods of gene therapy treatment, which are already available for some patients with muscular dystrophies, including the more controversial topics surrounding the price of the most modern drugs.^21^

### Teacher and Student Evaluation

From the perspective of a teacher with many years of experience, an increase in students’ interest was observed when working on the tasks described above, as well as a better fulfillment of teaching objectives in the given topics compared to the situation before the introduction of this innovation.

In the anonymous end-of-course survey of student opinions on seminar content that incorporated the new tasks, students consistently rated the tasks positively. 15% of students (20 out of 135) who attended the seminars wrote a comment specifically mentioning the new tasks. Students reported a better understanding of the connection between cellular mechanisms and clinical symptoms, greater confidence in diagnostic reasoning, and an increased ability to search and interpret scientific literature.

Based on feedback on the innovative tasks by teachers and students, it was recommended to implement these tasks in other groups as well and an adjustment of the learning outcomes of the relevant seminars was proposed. An example of the change in learning outcomes is provided in Table 1.

**Table 1.** An Example of the Change in Learning Outcomes Based on the New Tasks

Original learning outcome	Revised learning outcome	Assessment/evidence
Explain the function of adhesion proteins	Explain the role of selectins and integrins during leukocyte extravasation	Student explains consequences of abnormal function of adhesion proteins during seminar discussion
Know the key phases of the leucocyte extravasation process	Predicts the consequences of disrupting key steps in leukocyte transmigration on cell behavior	In group discussion, the student predicts the consequences of the affected phase and provides a biological justification
List the main manifestations of the so-called leukocyte adhesion deficiency syndromes	Infer likely clinical manifestations from dysfunction of selected adhesion proteins	Student proposes clinically plausible symptoms supported by biological reasoning
Use literature to find clinical data	Locate, appraise and use relevant scientific literature to verify hypotheses	Student identifies appropriate sources and uses them to support or revise predictions
Know the concept of genocopy	Explains why malfunctions of different proteins can result in similar clinical manifestations	Student identifies possible different proteins whose disorder could result in identical manifestations

## Discussion

Teaching cell biology in medical schools often risks becoming abstract, leaving students unsure of its relevance to clinical practice. Our approach—asking students to infer clinical symptoms from molecular dysfunction—helps bridge this gap by linking basic science content directly to diagnostic reasoning. This strategy aligns with broader efforts in medical education to anchor foundational sciences within realistic clinical frameworks.

A substantial body of research shows that explicitly integrating clinical context into basic science teaching enhances both comprehension and long-term retention. One widely used framework is clinical correlations, which encompass methods ranging from brief contextual examples to small-group problem solving. These approaches improve interest, communication, retention, and basic-to-clinical transfer by helping students situate molecular processes within medical practice.^22^ Such evidence supports our finding that even short, well-structured tasks can substantially shift students’ perceptions of relevance.

Another strategy with demonstrated benefit is Case-Based Collaborative Learning (CBCL) – a structured, interactive method that merges basic science and clinical reasoning. CBCL improves inductive reasoning, stimulates curiosity, and enhances integrative thinking by having students co-construct understanding from cases.^23^ Our symptom-inference tasks share similar pedagogical ideas: both require students to apply biochemical and cellular mechanisms to clinical situations, thereby reinforcing the usefulness of cell biology knowledge in decision-making contexts.

The short tasks we introduced engaged students, reinforced understanding of cellular processes, and stimulated early diagnostic thinking. Unlike extended projects or later-stage clinical cases described in published studies,^24-26^ our method provides brief, scalable, and adaptable activities suitable for first- and second-year courses. The revised learning outcomes, which were developed based on the evaluation of the tasks, emphasize higher-order cognitive processes such as explaining, predicting, inferring, and verifying. This shift reflects the principles of constructive alignment and better aligns with the diagnostic reasoning expected of future physicians.^8^

The evaluation of the benefits of the described new tasks was based on qualitative assessments by experienced teachers and feedback from students. The main limitation of the presented work is therefore the absence of a deeper evaluation of the effect of the new tasks on students that can be measured using quantitative methods between intervention and non-intervention groups. The lack of a formal calculation and justification of the sample size selected can also be considered a limitation.

## Conclusion

Teachers and students valued the relevance of these tasks and reported greater motivation and confidence in connecting molecular biology to clinical symptoms. The analysis of tasks resulted in modifications of learning outcomes towards greater emphasis on the ability to draw conclusions from cellular processes. The exercises also promoted transferable skills such as literature searching and critical appraisal. While our evaluation relied on surveys rather than controlled measures, the consistent positive feedback suggests that clinically anchored tasks can enrich preclinical teaching. Future studies should assess long-term knowledge retention and diagnostic performance.

## Supplemental Material

Supplemental Material - Estimating Clinical Manifestations in Cell Biology Teaching is a Suitable Active Element for Teaching Beginning Medical Students Supplemental Material for Estimating Clinical Manifestations in Cell Biology Teaching is a Suitable Active Element for Teaching Beginning Medical Students by Pavel Dvorak and Zbynek Tonar in Journal of Medical Education and Curricular Development.

Supplemental Material - Estimating Clinical Manifestations in Cell Biology Teaching is a Suitable Active Element for Teaching Beginning Medical Students Supplemental Material for Estimating Clinical Manifestations in Cell Biology Teaching is a Suitable Active Element for Teaching Beginning Medical Students by Pavel Dvorak and Zbynek Tonar in Journal of Medical Education and Curricular Development.

## Data Availability

All data generated or analyzed during this study are included in this published article [and its supplementary information files].*
