# Supplementary material for: Estimating Clinical Manifestations in Cell Biology Teaching is a Suitable Active Element for Teaching Beginning Medical Students
Source: J Med Educ Curric Dev. 2026 Sep 2;13:23821205261478406. doi: 10.1177/23821205261478406 (PMC13539034; doi:10.1177/23821205261478406)
Supplement: Supplementary file 1 [file sj-pdf-1-mde-10.1177_23821205261478406.pdf]

Supplementary Material 1

Questions for the student questionnaire seeking feedback on the course content after its completion

For the answers to the first 5 questions, use a numerical scale from number 1 – the worst result – to number 10 – the best result.

1. What number would you rate your overall impression of the course you completed?

2. What number would you rate the appropriateness of the cell biology topics included?

3. What number would you rate the chosen teaching methods for the theoretical parts of the course?

4. What number would you rate the chosen tasks for the practical parts of the course?

5. What number would you rate the benefit of the course for your further personal development?

Answer the following questions with a short written answer.

6. What do you see as the main benefits of the theoretical parts of the course?

7. What do you see as the main weaknesses of the theoretical parts of the course?

8. What do you see as the main benefits of the practical parts of the course?

9. What do you see as the main weaknesses of the practical parts of the course?

10. Which teaching moment interested you the most?

11. Which teaching elements/tasks do you think would most deserve to be innovated and why?
